# Eugenol‐Derived Piperazine Mannich Bases as Selective Inhibitors of Mayaro Virus: In Vitro Antiviral Evaluation and Mechanistic Insights

**DOI:** 10.1002/jmv.71158

**Published:** 2026-09-21

**Authors:** Adriana Cotta Cardoso Reis, Amália Luíza de Sousa da Silva, Camila Portruneli, Augusto Vieira Pontes Silva, Saulo Fehelberg Pinto Braga, Igor Rodrigues Lapa, Thiago Belarmino de Souza, Lucas Lopardi Franco, Diogo Teixeira Carvalho, Geraldo Célio Brandão

**Affiliations:** ^1^ Pharmacy School Federal University of Ouro Preto Ouro Preto Brazil; ^2^ Department of Pharmaceutical Sciences Federal University of Alfenas Alfenas Brazil

**Keywords:** antiviral activity, Arboviruses, Chikungunya virus, eugenol derivatives, Mayaro virus, piperazine Mannich bases

## Abstract

Arboviruses represent an ongoing global health challenge, particularly in tropical regions where diseases caused by *Chikungunya virus* (CHIKV) and *Mayaro virus* (MAYV) continue to emerge. Given the absence of licensed antiviral therapies, the identification of effective small molecules remains critical. This study evaluated sixteen eugenol‐ and dihydroeugenol‐derived piperazine Mannich bases for antiviral activity against CHIKV, MAYV, and *Zika virus* (ZIKV) in Vero cells. Cytotoxicity was assessed using the MTT assay, and antiviral activity was quantified through median effective concentration (EC_50_) and selectivity index (SI) values. Time‐dependent cytopathic effect inhibition, plaque reduction assays, virucidal assays and docking were performed to characterize the mechanism of action. Three compounds (**D3**, **D7**, and **E7**) exhibited selective antiviral activity. Notably, compound **D7** demonstrated low‐micromolar anti‐MAYV inhibition (EC_50_ = 14.43 μM; SI > 16.6), surpassing ribavirin by more than thirty‐fold in relative potency. Time‐dependent assays revealed progressive viral titer reductions of up to 3 log_10_ PFU/mL over 48 h. Virucidal assays confirmed that **D7** does not directly inactivate viral particles. These findings identify **D7** as a promising antiviral lead compound and provide a foundation for further mechanistic, optimization, and in vivo studies aimed at developing anti‐alphavirus therapeutics.

## 1 Introduction

Arthropod‐borne viruses (arboviruses) represent a persistent and expanding global health challenge, particularly in tropical and subtropical regions where ecological conditions favor vector proliferation [1]. Transmission to humans occurs primarily through infected mosquitoes of the genera Aedes, Culex, and Haemagogus, enabling sustained zoonotic and urban transmission cycles [2]. These complex transmission dynamics facilitate rapid geographic spread and recurrent outbreaks, especially in regions with high vector density and limited public health infrastructure [3].

Over 150 arboviruses are recognized as human pathogens, many of which are responsible for epidemic or endemic disease. The majority of clinically relevant species belong to four principal genera: Orthoflavivirus (family Flaviviridae), Alphavirus (family Togaviridae), Orthobunyavirus (family Peribunyaviridae), and Phlebovirus (family Phenuiviridae) [4]. Collectively, these viruses account for an estimated 100 million symptomatic infections annually. Among the most impactful are Dengue virus, ZIKV, Yellow fever virus, Japanese encephalitis virus, and West Nile virus, as well as the arthritogenic alphaviruses CHIKV, MAYV, and Sindbis virus [4, 5].

In recent years, the epidemiological landscape of arboviral diseases in the Americas has shifted considerably. The expansion of CHIKV since its introduction into the Western Hemisphere and the increasing detection of MAYV in urban and peri‐urban settings have raised concerns regarding potential co‐circulation and misdiagnosis. MAYV, historically confined to sylvatic transmission cycles in the Amazon basin, has demonstrated increasing outbreak frequency and geographic expansion, highlighting its emergence as a neglected but potentially significant tropical pathogen. Clinically, MAYV infection resembles chikungunya fever, characterized by febrile illness accompanied by debilitating polyarthralgia that may persist for months [6, 7, 8].

Despite the substantial morbidity associated with these infections, therapeutic options remain limited [9, 10]. Currently, there are no licensed specific antiviral drugs targeting most arboviruses, and vaccine availability is restricted to a small number of flaviviral infections. Clinical management is therefore largely supportive, emphasizing the urgent need for novel antiviral agents capable of reducing viral replication and disease burden [11, 12, 13].

Natural products and their derivatives continue to play a central role in drug discovery, particularly in the identification of structurally diverse scaffolds suitable for rational optimization. Among these, phenylpropanoids have attracted considerable attention due to their broad biological activity spectrum and synthetic versatility. Eugenol, a major constituent of clove essential oil, represents a privileged phenylpropanoid scaffold with well‐documented antimicrobial, anti‐inflammatory, and antiparasitic properties. Structural modification of eugenol and related analogues, such as dihydroeugenol and isoeugenol, enables the generation of chemically diverse derivatives with enhanced pharmacological profiles [11, 12, 13].

The incorporation of heterocyclic moieties into natural‐product‐inspired frameworks is a widely employed strategy to improve biological activity and pharmacokinetic properties. In this context, piperazine is a pharmacologically relevant fragment frequently found in bioactive molecules, including antiviral agents, due to its capacity to modulate solubility, binding interactions, and molecular conformation. Mannich base formation further expands structural diversity and has been associated with improved biological activity across multiple therapeutic areas [14, 15, 16, 17, 18].

Our research group has systematically explored phenylpropanoid scaffolds for the development of bioactive compounds with antibacterial, antifungal, and antiparasitic properties [14, 15, 16, 17]. More recently, we reported eugenol‐derived benzoxazole derivatives exhibiting promising anti‐arboviral potential [18]. Building upon this platform, a chemical library of eugenol‐ and dihydroeugenol‐derived piperazine Mannich bases was previously synthesized and characterized, initially investigated for antimycobacterial activity.

Given the structural features of these compounds and the recognized antiviral potential of heterocyclic Mannich derivatives, we hypothesized that this library could contain molecules capable of interfering with arboviral replication. Therefore, the present study aimed to evaluate these derivatives against clinically relevant tropical arboviruses, including CHIKV, MAYV, and ZIKV, and to identify potential hit compounds for further antiviral development.

## 2 Materials and Methods

### 2.1 Synthesis of Piperazine Derivatives

**Scheme 1:**
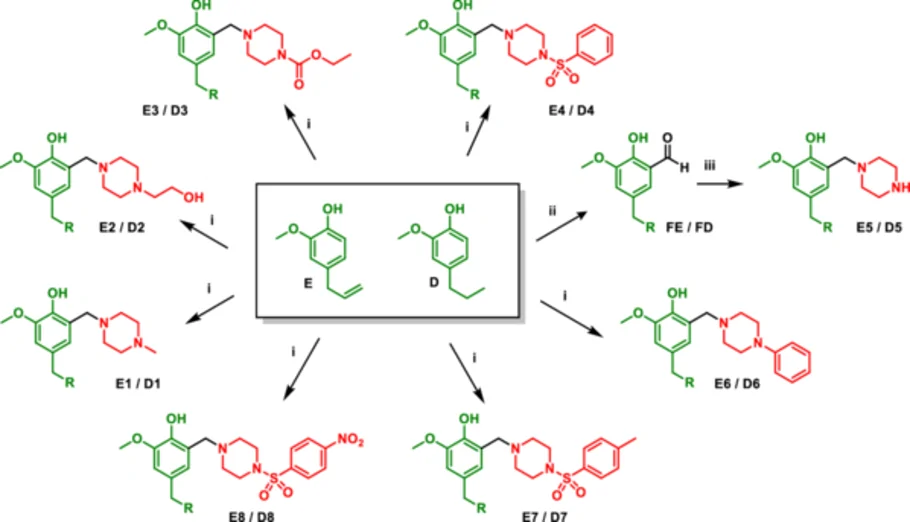
– Synthesis route for compounds E1‐E8 and D1‐D8. Reaction conditions: (i) *N‐*substituted piperazine, formaldehyde, HCl cat., toluene, 95°C; (ii) hexamine, glacial AcOH, 140°C and then HCl aq.; (iii) piperazine, NaCNBH_3_, THF/MeOH, 50°C.

The piperazine Mannich base derivatives evaluated in this study were synthesized and fully characterized in our previous work [19]. Briefly, eugenol and dihydroeugenol were used as starting phenylpropanoid scaffolds for the preparation of their corresponding Mannich bases via a three‐component reaction involving formaldehyde and *N*‐substituted piperazines under acid catalyzed conditions with controlled heating (Scheme 1). The derivatives obtained from eugenol are designated as E1–E8, while those from dihydroeugenol are designated as D1–D8.

For compounds E5 and D5, synthesis was performed through reductive amination of the corresponding formylated intermediates (formyl‐eugenol and formyl‐dihydroeugenol) using piperazine in the presence of sodium cyanoborohydride as a reducing agent. All compounds were previously characterized by spectroscopic techniques and analytical methods confirming structural integrity and purity [19].

### 2.2 Cell Lines and Virus Strains

African green monkey kidney epithelial cells (American Type Culture Collection CCL‐81; Vero) were maintained in Dulbecco's Modified Eagle's Medium (DMEM). Mosquito‐derived cells (American Type Culture Collection CRL‐1660; C6/36, Aedes albopictus) were cultured in Leibovitz's L‐15 medium. Both media were supplemented with 5% fetal bovine serum (FBS), penicillin–streptomycin (100 U/mL), and amphotericin B (5 µg/mL). Mammalian cells were maintained at 37°C in a humidified 5% CO2 atmosphere, whereas C6/36 cells were incubated at 28°C without CO2 supplementation.

The following viral strains were used: Orthoflavivirus zikaense (ZIKV, strain PE243/2015), Alphavirus chikungunya (CHIKV, strain S27‐African), and Alphavirus mayaro (MAYV, strain Acre27). ZIKV was propagated in C6/36 cells using L‐15 medium without FBS during viral adsorption. CHIKV and MAYV were propagated in Vero cells using serum‐free DMEM supplemented with antibiotics during infection.

Viral supernatants were harvested upon observation of cytopathic effect, clarified by centrifugation, aliquoted, and stored at −80°C. Viral titers were determined by 50% tissue culture infectious dose (TCID50/mL) [20] and plaque‐forming unit per milliliter (PFU/mL) assays according to standard protocols [21].

### 2.3 Cytotoxicity Assessment

Cell viability was evaluated using the 3‐(4,5‐dimethylthiazol‐2‐yl)−2,5‐diphenyltetrazolium bromide (MTT) colorimetric assay.

Vero cells (1 × 10^4^ cells/well) were seeded in 96‐well plates and allowed to adhere overnight. Test compounds were dissolved in dimethyl sulfoxide (final solvent concentration < 0.5%) and serially diluted in culture medium. Cells were exposed to compounds for 72 h.

After incubation, MTT solution (2 µg/mL in phosphate‐buffered saline) was added and plates were incubated for 2 h at 37°C. Formazan crystals were solubilized, and absorbance was measured at 490 nm using a microplate reader.

The 50% cytotoxic concentration (CC50) was determined by nonlinear regression analysis using GraphPad Prism (version 8.0.2). Results represent the mean ± standard deviation of three independent experiments performed in triplicate [18].

### 2.4 In Vitro Antiviral Activity Assay

Antiviral activity was assessed by measuring protection against virus‐induced cytopathic effect (CPE).

Vero cells (1 × 10^4^ cells/well) were infected with ZIKV (1 × 10^3^ TCID_50_/mL), CHIKV (1 × 10^3^ TCID_50_/mL), or MAYV (1 × 10^4^ TCID_50_/mL). After viral adsorption, cells were treated with eight serial dilutions of each compound at non‐cytotoxic concentrations and incubated for 72 h.

Cell viability was subsequently quantified by MTT assay as described above. The median effective concentration (EC50) was defined as the concentration required to reduce virus‐induced cytopathic effect by 50% [22, 23].

Ribavirin was used as a reference antiviral control for ZIKV and MAYV assays [24, 25], while amantadine was used for CHIKV assays [26]. The selectivity index (SI) was calculated as CC50/EC50. Relative potency was determined by comparing the EC50 of the reference compound to that of each test derivative.

### 2.5 Time‐Dependent Cytopathic Effect Inhibition Assay

To further characterize antiviral activity, a time course CPE inhibition assay was performed for the most active compound (piperazine derivative D7) against MAYV.

Vero cells (5 × 10^5^ cells/well) were seeded in 12‐well plates and infected with MAYV at a multiplicity of infection (MOI) of 0.1. Cells were treated with D7 at 23.91 µM. Four experimental groups were established: (i) uninfected cells (cell control), (ii) uninfected treated cells (compound control), (iii) infected untreated cells (virus control), and (iv) infected treated cells (antiviral condition) [18].

Cell morphology was documented at 24, 36, and 48 h post‐infection using phase‐contrast microscopy. At each time point, culture supernatants were collected and stored at −80°C until further analysis. Viral titers were subsequently determined by plaque assay in Vero cells as described by Dulbecco [21]. Briefly, supernatants from both the viral control and antiviral treatment groups were subjected to tenfold serial dilutions in serum‐free DMEM and inoculated onto Vero cell monolayers. Following a 1 h adsorption period at 37°C in a humidified atmosphere containing 5% CO_2_, the inoculum was removed and replaced with semisolid DMEM overlay medium. The plates were then incubated at 37°C for 3 days, after which viral plaques were quantified to determine infectious viral titers [21].

### 2.6 Virucidal Activity Assay

To determine whether D7 directly inactivates viral particles, a virucidal assay was performed. Mayaro virus (MOI = 10) was incubated with D7 (23.91 µM) for 1 h at 37°C. An untreated viral suspension (MOI = 10) served as control. After incubation, samples were serially diluted and subjected to plaque assay in Vero cells, in triplicate, as previously described. Viral titers were expressed as PFU/mL [27].

### 2.7 Statistical Analysis

Data are presented as mean ± standard deviation. Differences in viral titers (Log_10_ PFU/mL) between treated and untreated groups were analyzed using Student's t‐test in GraphPad Prism (version 8.0.2). A *p*‐value < 0.05 was considered statistically significant.

### 2.8 Molecular Modeling Studies

The prediction of the complex three‐dimensional structures of molecular targets and their affinity was performed using the RowanSci platform (https://labs.rowansci.com/) from the amino acid sequences obtained from UniProt (https://www.uniprot.org/) and the SMILES code of derivative **D7**. The analyses were conducted in protein‐ligand co‐folding mode using Boltz‐2 as the model in quintuplicate. To optimize the accuracy of the results, the tests were performed with Multiple Sequence Alignment (MSA) mode activated. For each target, ten samples were requested and the pTM and pLDDT parameters were analyzed for each prediction.

Traditional molecular docking studies were performed using the GOLD program (CCDC) [28]. For the NSP3 protein, docking was performed using the structure obtained from the Protein Data Bank (PDB:5IQ5). For the remaining targets, the analyses were performed using the structures derived from Boltz‐2 prediction. Three‐dimensional structure of derivative D7 and lobaric acid was generated with the gen3D function of the OpenBabel program [29] using the best option. Subsequently, a conformational search was performed using obconformer (25 conformations, 1000 minimization steps), followed by minimization of the best conformation (50,000 steps of the steepest descent algorithm with a convergence criterion of 10^−7^, using the MMFF94 force field) [29]. A total of 100 genetic algorithm runs were performed in a search area defined as 12 Å from the coordinates of the best ligand pose predicted by Boltz‐2, using ChemPLP as the scoring function. A minimum of 150,000 genetic algorithm operations were requested, and the ten best solutions were saved for analysis. Finally, the conformations and interactions of the complex were analyzed in Discovery Studio Visualizer (Dassault Systèmes).

Sequence alignment between MAYV and CHIKV NSP1 sequences was performed using BLAST, with default parameters, to identify equivalent residue positions [30]. Structural superposition of the corresponding NSP1 models and comparative analysis of the aligned residues and predicted binding poses were carried out in PyMOL (The PyMOL Molecular Graphics System, Version 3.0.5 Schrödinger LLC.).

Molecular dynamics analyses were performed using the GROMACS software [31]. Simulations were carried out starting from the best pose obtained in the molecular docking study for the NSP1 enzyme. Ligand topology was generated using the Acpype program, based on the GAFF2 force field, whereas protein topology was generated using the AMBER03 force field [32, 33]. Subsequently, a protein‐ligand complex file was built by combining the topology data into a single file. The system was placed in a dodecahedral simulation box, with a minimum distance of 1.2 nm between the protein‐ligand complex and the box edges. Solvation was performed using SPC216 water model molecules, followed by system neutralization through the addition of Na^+^ and Cl^−^ ions. The complex was initially subjected to energy minimization using the steepest descent algorithm for 50,000 steps or until a maximum force of 100.0 kJ/mol was reached, followed by equilibration steps under NVT (constant number of particles, volume, and temperature) and NPT (constant number of particles, pressure, and temperature) conditions, each lasting 1 ns. During these steps, the V‐rescale thermostat (310 K) and the C‐rescaled barostat (1 bar) were employed. After equilibration, molecular dynamics simulations were performed for 200 ns [34]. Results were analyzed using PyMOL and R Studio software.

## 3 Results and Discussion

### 3.1 Design and Synthesis of Piperazine Derivatives

Two series of piperazine Mannich bases derived from eugenol (E1–E8) and dihydroeugenol (D1–D8) were synthesized through a three‐step synthetic route (Scheme 1). The modular approach enabled systematic variation of piperazine substitution patterns while maintaining the phenylpropanoid core structure. This design strategy was specifically oriented to evaluate how saturation of the allylic side chain influences antiviral activity against alphaviruses.

The synthetic protocol afforded target compounds with yields in the range of 13%–84% [19], sufficient for comprehensive biological evaluation. Structural diversification between the E‐series (unsaturated) and d‐series (saturated) was intentionally implemented to probe structure activity relationships regarding lipophilicity, conformational flexibility, and potential metabolic stability.

### 3.2 Cytotoxicity Assessment

Prior to antiviral screening, the cytotoxicity profile of all sixteen derivatives was determined in Vero cells using the MTT assay. Eight compounds (50%) exhibited no measurable cytotoxicity at the highest concentration tested (602.05 µM for d‐series precursors; 609.45 µM for E‐series). The remaining compounds displayed CC_50_ values ranging from 199.55 to 358.02 µM (Table 1).

**Table 1:** In vitro cytotoxicity (CC_50_) and antiviral activity (EC_50_) assessments of synthesized compounds against ZIKV, CHIKV, and MAYV in Vero cells, determined by the MTT assay after 72 h of treatment. Data are expressed as mean ± standard deviation (*n* = 3). Selectivity index (SI) and relative potency (RP) values are also presented, highlighting the antiviral efficacy and cytotoxic profiles of the evaluated compounds.

Substance	VERO CC_50_ µg/mL	ZIKV a EC_50_ µg/mL	SI^b^	RP^c^	CHIKV d EC_50_ µg/mL	SI	RP	MAYV e EC_50_ µg/mL	SI	RP
D	> 602.05	NA^f^	– g	—	NA	—	—	NA	—	—
D1	300.25 ± 2.03	NA	—	—	NA	—	—	NA	—	—
D2	230.46 ± 1.46	NA	—	—	NA	—	—	NA	—	—
D3	259.34 ± 1.94	NA	—	—	**165.05** ± **1.37**	**1.6**	**2.5**	NA	—	—
D4	> 247.42	NA	—	—	NA	—	—	NA	—	—
D5	276.81 ± 1.57	NA	—	—	NA	—	—	NA	—	—
D6	> 293.93	NA	—	—	NA	—	—	NA	—	—
D7	> 239.12	NA	—	—	NA	—	—	**14.43** ± **1.41**	**> 16.6**	**29.9**
D8	199.55 ± 2.50	NA	—	—	NA	—	—	NA	—	—
E	> 609.45	NA	—	—	NA	—	—	NA	—	—
E1	358.02 ± 3.53	NA	—	—	NA	—	—	NA	—	—
E2	254.09 ± 1.92	NA	—	—	NA	—	—	NA	—	—
E3	228.97 ± 1.61	NA	—	—	NA	—	—	NA	—	—
E4	> 248.66	NA	—	—	NA	—	—	NA	—	—
E5	306.56 ± 1.76	NA	—	—	NA	—	—	NA	—	—
E6	> 295.68	NA	—	—	NA	—	—	NA	—	—
E7	> 240.28	NA	—	—	**33.30** ± **1.55**	**> 7.2**	**12.6**	NA	—	—
E8	> 223.64	NA	—	—	NA	—	—	NA	—	—
Ribavirin (positive control)	1516.75 ± 1.20	433.24 ± 1.60	3.50	—	NA	—	—	431.24 ± 2.21	3.51	—
Amantadine (positive control)	561.26 ± 1.16	NT h	—	—	418.98 ± 1.70	1.34	—	NT	—	—

^a^ ZIKV: viral titer 1 × 10^3^ TCID_50_/mL.

^b^ SI (Selectivity Index): the ratio of CC_50_ to EC_50_.

^c^ RP (Relative Potency): ratio between positive control EC_50_ and active compound EC_50_.

^d^ CHIKV: viral titer 1 × 10^3^ TCID_50_/mL.

^e^ MAYV: viral titer 1 × 10^4^ TCID_50_/mL.

^f^ NA: Not active.

^g^ –: Not determined.

^h^ NT: Not tested.

**Figure 1:**
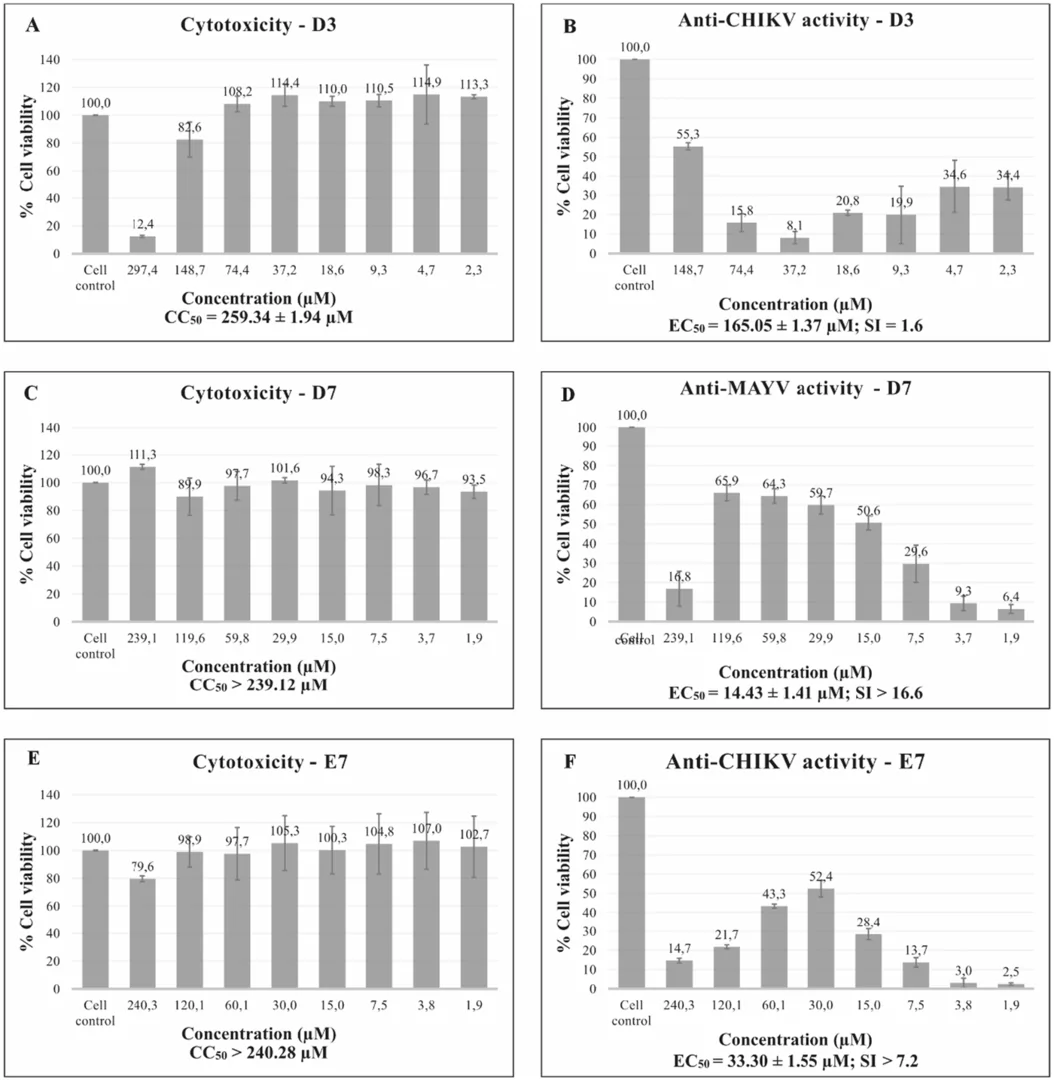
Dose–response curves for the in vitro cytotoxicity (CC_50_) and antiviral activity (EC_50_) of the active piperazine derivatives **D3** (A and B), **D7** (C and D), and **E7** (E and F) in Vero cells, determined by the MTT assay after 72 h of treatment (*n* = 3). Data are expressed as the mean ± standard deviation of cell viability relative to untreated cell control.

Notably, compounds D7 and E7 exhibited favorable safety profiles, maintaining cell viability above 90% after 72 h of exposure at concentrations up to 239.12 and 240.28 µM, respectively (Figure 1C,E). Similarly, **D3** maintained cell viability above 80% at 148.72 µM (Figure 1A). Overall, these results demonstrate that the antiviral effects observed for the active compounds were not attributable to nonspecific cytotoxicity, thereby supporting that the reduction in viral replication reflects a genuine antiviral effect rather than compound‐induced loss of cell viability.

### 3.3 Antiviral Screening against Arboviruses

The antiviral potential of the synthesized derivatives was evaluated against CHIKV, MAYV, and ZIKV in Vero cells using MTT‐based cytoprotecting assays (Table 1).

Among the evaluated compounds, **D3** and **E7** exhibited antiviral activity against CHIKV, with EC_50_ values of 165.05 ± 1.37 µM and 33.30 ± 1.55 µM, respectively (Figure 1B,F). However, despite its measurable antiviral effect, **D3** displayed a low selectivity index (SI = 1.6), indicating that its antiviral activity occurred at concentrations close to those associated with cytotoxicity. Consequently, **D3** cannot be considered a promising lead candidate. In contrast, **E7** exhibited greater antiviral potency together with moderate selectivity (SI > 7.2), indicating a more favorable antiviral profile against CHIKV, although its selectivity remains below the threshold generally considered desirable for lead compounds. The piperazine derivative **D7**, on the other hand, showed low‐micromolar antiviral activity and selective inhibition of MAYV replication (EC_50_ = 14.43 ± 1.41 µM; SI > 16.6; Figure 1D), while exhibiting no significant activity against CHIKV or ZIKV. None of the evaluated compounds demonstrated meaningful antiviral activity against the flavivirus ZIKV, with EC_50_ values exceeding the highest concentration tested (Table 1).

The selectivity index (SI) of **D7** (> 16.6) indicated a substantial therapeutic window. Most significantly, **D7** was approximately 30‐fold more potent than ribavirin (EC_50_ = 431.24 ± 2.21 µM) under identical experimental conditions, representing a significant improvement over ribavirin, the current reference compound with limited clinical utility against alphaviruses.

The differential activity profiles observed between the E‐series (eugenol‐derived) and d‐series (dihydroeugenol‐derived) compounds suggest that saturation of the allylic side chain favors antiviral activity against alphaviruses. This finding aligns with previous reports indicating that hydrogenation of the propenyl group in eugenol derivatives can enhance biological stability and reduce metabolic oxidation, potentially improving target engagement [35, 36].

The superior activity of D7 compared to its eugenol counterpart (E7 inactive against MAYV) supports the hypothesis that the saturated propyl chain confers optimal conformational and lipophilic properties for interaction with viral targets. The piperazine moiety, a privileged scaffold in antiviral drug discovery, likely contributes to activity through potential interactions with viral proteins involved in replication complex formation [35, 36].

The marked selectivity of D7 for MAYV over CHIKV and ZIKV is particularly noteworthy. While both MAYV and CHIKV are alphaviruses with similar genomic organization, the 30‐fold difference in potency (D7 active against MAYV but not CHIKV) suggests specific targeting of MAYV encoded functions rather than broad alphavirus inhibition. This selectivity profile contrasts with compounds like favipiravir, which exhibits pan‐alphavirus activity through RNA polymerase inhibition [37, 38].

Based on these results, the piperazine derivative **D7** exhibited the lowest EC_50_ value and the highest SI against MAYV, highlighting it as the most promising compound. Therefore, **D7** was selected for further antiviral investigation through additional in vitro assays and in silico analyses.

### 3.4 Time‐Dependent Inhibition of MAYV Replication

To characterize the kinetics of antiviral activity, compound **D7** was evaluated in a time‐dependent cytopathic effect (CPE) inhibition assay against MAYV (MOI = 0.1).

**Figure 2:**
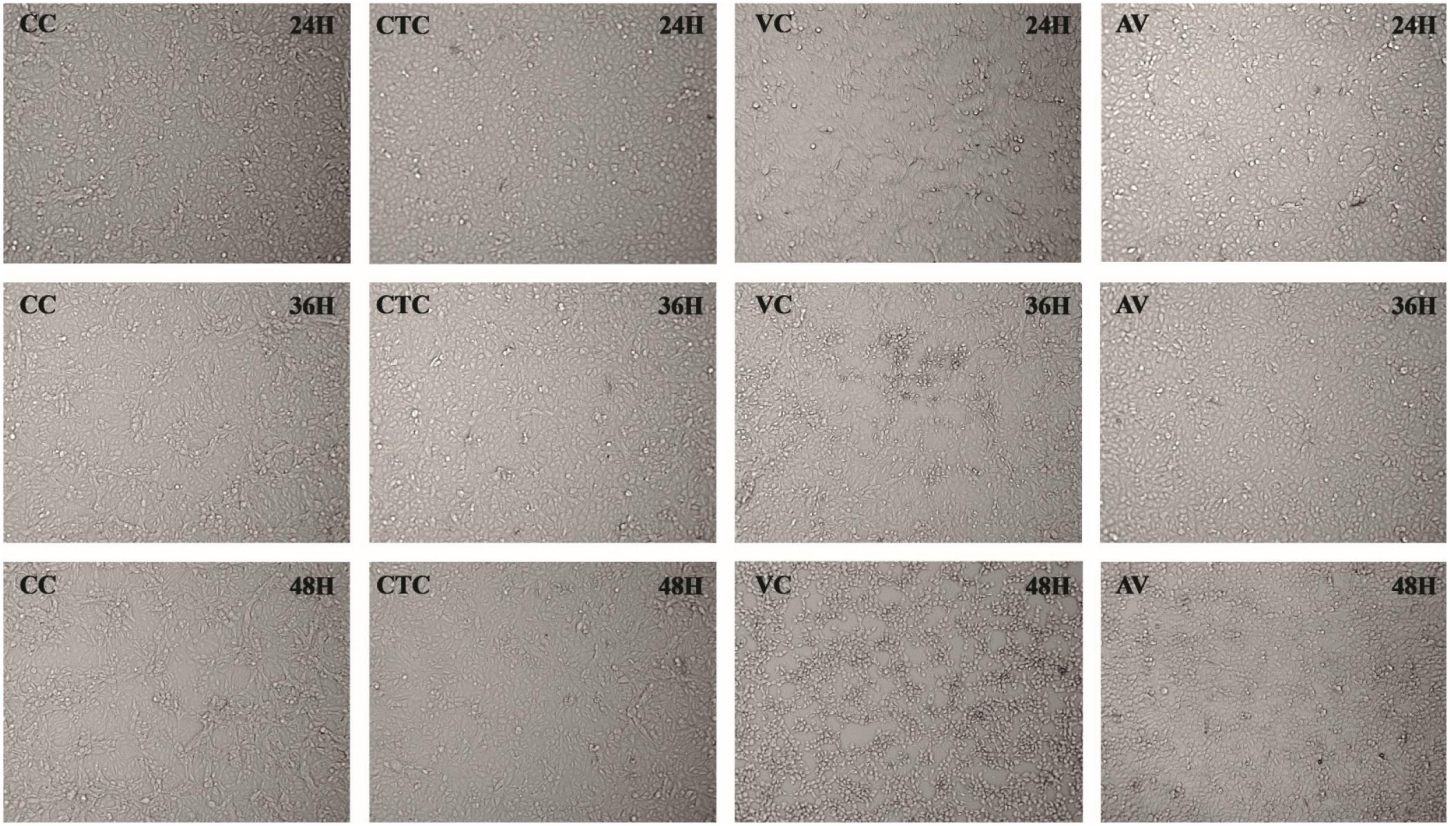
Time‐dependent inhibitory cytopathic effect of the synthesized compound **D7** against MAYV. Vero cells were infected with MAYV (MOI = 0.1), treated with compound **D7** (23.9 µM), and photographed 24‐, 36‐ and 48‐ hour post‐infection. Representative images were obtained using ZEN 2.3 Lite software at 100× magnification. AV, Antiviral activity of **D7** (23.9 µM).µM); CC, Cell Control; CTC, Cytotoxicity Control of **D7** (23.9 µM); VC, Viral Control.

Microscopic analysis revealed progressive cytopathic alterations in untreated infected cells, including cell rounding, detachment, and monolayer disruption. In contrast, **D7**‐treated cultures (23.9 µM) showed marked preservation of cellular morphology at all time points evaluated (24, 36, and 48 h post‐infection) (Figure 2).

Viral yield reduction assays demonstrated time‐dependent suppression of infectious virus production: 24 h.p.i.: 1.0 log_10_ reduction (3.20 × 10^7^ vs. 4.44 × 10^8^ PFU/mL); 36 h.p.i.: 2.7 log_10_ reduction (2.165 × 10^10^ vs. 1.05 × 10^13^ PFU/mL); 48 h.p.i.: 3.0 log_10_ reduction (1.58 × 10^11^ vs. 1.535 × 10^14^ PFU/mL).

**Figure 3:**
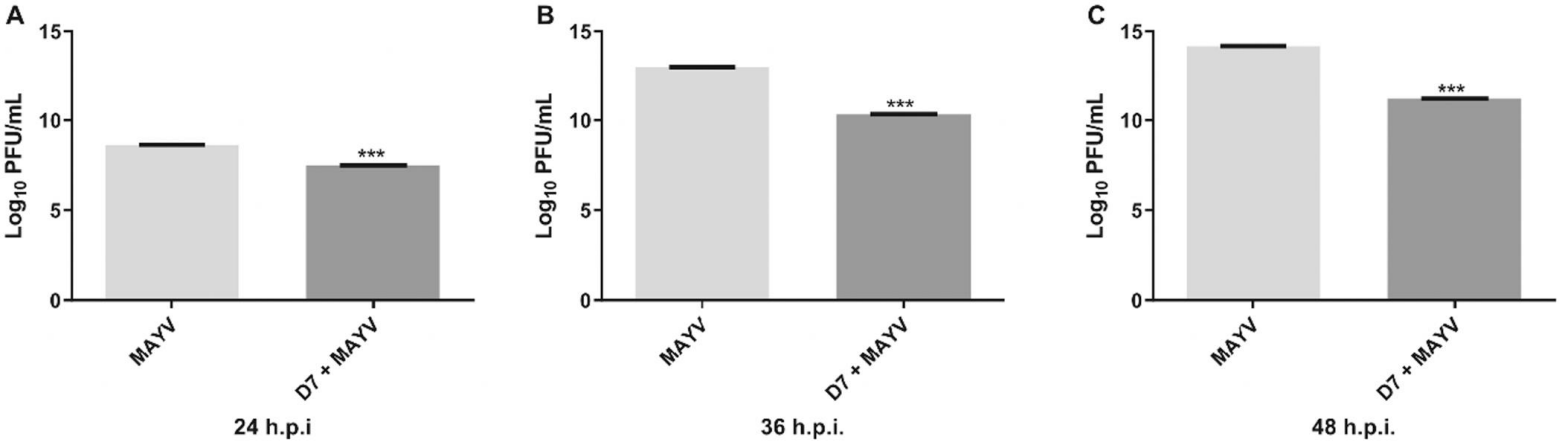
Viral yield quantification by plaque reduction assay (log10 PFU/mL) following the inhibitory CPE assay at 24 h post‐infection (h.p.i., A), 36 h.p.i (B), and 48 h.p.i (C) Vero cells infected with MAYV (MOI = 0.1) and treated with synthesized compound D7 (23.9 µM) or left untreated (viral control). The plaque reduction assay was performed using 10‐fold serially diluted treated with D7 along with respective untreated virus control. Titers of MAYV were determined and the PFU/mL reduction with respect to virus control was calculated. Experiments were carried out in duplicate, and statistical analysis was conducted using Student's *t*‐test with a 95% confidence interval. MAYV: viral control (MOI = 0.1); D7 + MAYV: cell monolayers infected with MAYV (MOI = 0.1) and treated with synthesized compound D7 (23.9 µM) at 24‐, 36‐, and 48‐h post infection, ***: *p* < 0.05.

These data confirm sustained antiviral activity throughout the viral replication cycle and suggest interference with intracellular replication stages rather than transient or early‐entry inhibition (Table 2, Figure 3).

**Table 2:** Viral yield quantification by plaque reduction assay (PFU/mL) following the inhibitory CPE assay at 24‐, 36‐, and 48‐h post‐infection in Vero cells infected with MAYV (MOI = 0.1) and treated with synthesized compound D7 (23.9 µM) or left untreated (viral control).

	Time post infection	Titration media (PFU/mL)	Log_10_ PFU/mL
MAYV viral control	24 h	4.44 × 10^8^	8.635
**D7** + MAYV		3.20 × 10^7^	7.505
MAYV viral control	36 h	1.05 × 10^13^	13.02
**D7** + MAYV		2.165 × 10^10^	10.335
MAYV viral control	48 h	1.535 × 10^14^	14.185
**D7** + MAYV		1.58 × 10^11^	11.20

### 3.5 Virucidal Activity Assessment

To confirm previous hypothesis and to determine whether **D7** exerts direct virucidal effects, MAYV (MOI = 10) was pre‐incubated with the compound (23.9 µM) at 37°C, for 1 h prior to infection of Vero cells.

Pre‐treatment did not significantly reduce viral titers compared to untreated virus control [5.67 vs. 5.91 log_10_ PFU/mL), with no statistically significant difference observed (*p *> 0.05), as shown in Table 3 and in Figure S1 (Supporting Information).

**Table 3:** – Evaluation of the virucidal activity of the piperazine derivative D7 (23.9 µM) against MAYV (MOI = 1 and MOI = 10) by plaque assay in Vero cells. Viral suspensions were pre‐incubated with D7 for 1 h at 37°C before infection, and viral titers were subsequently determined by plaque assay using 10‐fold serial dilutions. Untreated MAYV served as the negative control. Data are expressed as log_10_ PFU/mL. Experiments were performed in triplicate, and statistical significance was assessed using Student's *t*‐test (95% confidence interval).

	MOI	Titration media (PFU/mL)	Log_10_ PFU/mL
MAYV viral control	1	5.330 × 10^3^	3.740
**D7** + MAYV		5.327 × 10^3^	3.723
MAYV viral control	10	8.325 × 10^5^	5.91
**D7** + MAYV		4.995 × 10^5^	5.67

The absence of virucidal activity indicates that **D7** does not directly inactivate viral particles, suggesting that its antiviral effect occurs during post‐adsorption stages of the MAYV replication cycle. This observation is consistent with the results obtained in the time‐dependent inhibition assay, which demonstrated a reduction in viral yield of up to 3 log_10_ at 48 h post‐infection.

Together, these findings support a post‐entry mechanism of action for D7, likely involving interference with intracellular stages of the viral life cycle, potentially affecting: viral RNA replication complex formation, non‐structural protein processing and/or viral protein synthesis or assembly. This proposed mechanism is in agreement with previous reports describing piperazine‐containing antivirals that target viral replication machinery rather than viral entry processes [35].

MAYV represents an emerging arboviral threat in Latin America, with increasing reports of urban transmission and clinical presentations overlapping with chikungunya fever [39, 40]. The absence of licensed vaccines or specific antiviral therapies creates an urgent need for drug candidates with novel mechanisms of action. The identification of D7 addresses this gap by providing a structural novel lead compound with sub‐micromolar potency (EC50 = 14.43 µM) and favorable cytotoxicity profile (CC50 > 239 µM).

The 30‐fold improvement in potency relative to ribavirin, combined with the favorable safety index, positions **D7** as a promising candidate for further optimization through medicinal chemistry approaches. Structure activity relationship studies focusing on the piperazine substitution pattern and phenolic modifications may yield derivatives with enhanced potency and druglike properties.

### 3.6 Molecular Modeling

Based on results of biological assays suggesting antiviral activity at intracellular replication stages, potential targets for the active derivative D7 were investigated in silico. The non‐structural proteins NSP1, NSP2, NSP3, and NSP4 were selected due to their roles in the viral replication complex, which coordinates the synthesis and processing of viral RNA [41]. We decided to not include structural proteins, including the capsid, in this screening strategy. Although virucidal and time‐dependent CPE inhibition assays indicated an intracellular mode of action, this alone does not distinguish between replication‐complex and structural proteins, both of which act post‐entry. Selection was therefore also guided by druggability, as NSP1‐NSP4 possess relatively well‐defined catalytic or nucleotide‐binding pockets amenable to structure‐based virtual screening, whereas structural proteins act largely through extended protein‐protein and protein‐nucleic acid interfaces, representing challenging targets for docking strategies [42].

Unfortunately, among the evaluated targets, only the macrodomain of NSP3 has available experimental structural data (PDB: 5IQ5), involved in replication and virulence [43]. Thus, protein structures in complex with D7 were obtained using Boltz‐2 predictions based on their FASTA sequences available on UniProt. The limited structural data available for Mayaro virus non‐structural proteins limits the investigation of potential ligand binding modes. To this context, artificial intelligence‐based approaches such as AlphaFold, ESMFold, and Boltz have become valuable alternatives for protein structure prediction [44]. Boltz‐2, an improved version of its predecessor Boltz‐1, is an open‐source code based on AlphaFold3, capable of predicting protein‐ligand complex structures and ligand binding probabilities [45].

Among the predicted models, NSP3 exhibited the lowest confidence scores, with a predicted template modeling (pTM) score of 0.67 and a predicted local distance difference test (pLDDT) score of 0.626. In contrast, NSP1, NSP2, and NSP4 showed pTM and pLDDT values above 0.8.

Despite its utility in structure prediction, ligand affinity estimation by Boltz‐2 remains less validated than classical molecular docking approaches [46]. Thus, we decided to evaluate the affinity of the active compound D7 on the molecular targets using classical docking using the well‐established GOLD software. Since the NSP3 protein exhibited low‐quality scores in the structure predicted by Boltz‐2, docking on this target was performed using the structural data available in the Protein Data Bank, whereas for the remaining proteins, docking was performed on the structures predicted by Boltz‐2.

Based on docking scores, NSP1 exhibited the highest affinity for D7, with a score of 92.31, significantly higher than those obtained for the other proteins evaluated (77.91, 69.64, and 73.12 for NSP2, NSP3, and NSP4, respectively).

**Figure 4:**
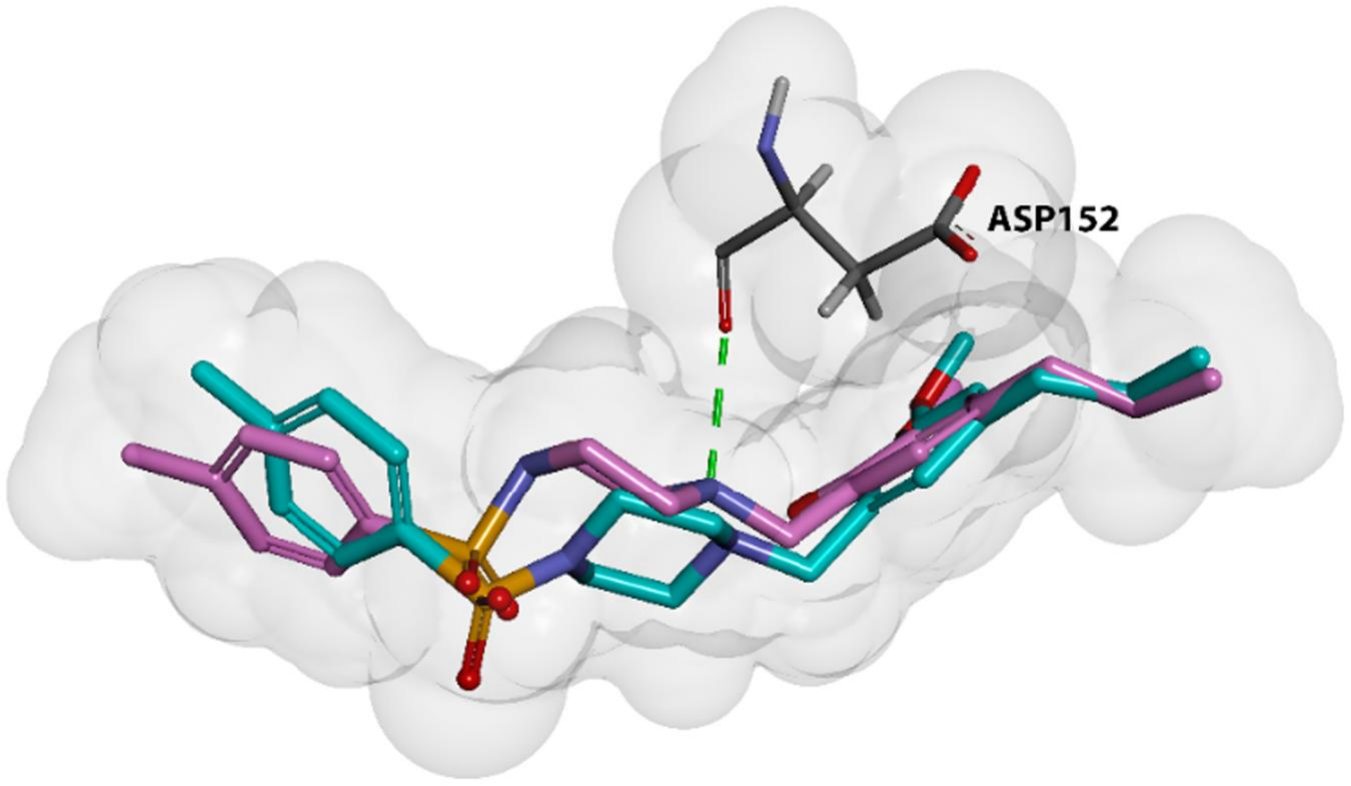
Best pose of the active derivative **D7** predicted by GOLD (pink) and Boltz‐2 (cyan). Dashed lines represent the hydrogen bond interaction between the nitrogen atom of the piperazine ring and the Asp152 residue. The active site residues are represented by a white molecular surface.

On the other hand, Boltz‐2 predicted low binding probabilities of **D7** across all targets (0.301–0.498, on a 0–1 scale, where higher values indicate greater binding likelihood). Rescoring of these poses using the ChemPLP function, allowing local minimization, resulted in lower ChemPLP scores compared to those obtained from docking. Furthermore, a comparison of binding poses of NSP1 complexes showed that in the docking‐derived pose a conformational rearrangement of the piperazine ring allowed a hydrogen bond with Asp152 while the Boltz‐2–predicted pose did not favor this interaction (Figure 4). These observations suggest that differences in ligand conformation, particularly within the piperazine moiety, likely contribute to the improved ranking of docking‐generated poses. In CHIKV NSP1, the equivalent residue contributes to the SAM‐binding pocket of the methyltransferase domain, contacting the methionine moiety of the SAM cofactor [47]. This position was found to be structurally equivalent between MAYV and CHIKV NSP1 given sequence and structural alignment performed with both proteins.

Additionally, to further support the in silico prioritization of NSP1, we evaluated lobaric acid, a natural compound with experimentally validated inhibitory activity against CHIKV NSP1, possessing antiviral activity against CHIKV and SINV in cell culture [48]. Although other NSP1‐targeting antivirals have also been reported, such as selective estrogen receptor modulators (tamoxifen, 4‐hydroxytamoxifen, and clomifene) [49], lobaric acid shares partial structural similarity with D7, supporting its use as a positive control for the docking protocol. On the other hand, ribavirin, commonly used as a reference antiviral in alphavirus studies, was not chosen, as recent biochemical evidence indicates it does not directly inhibit NSP1 methyltransferase activity, instead acting through an indirect mechanism mediated by IMPDH inhibition and cellular GTP depletion [50]. While the Boltz‐2 affinity probability for MAYV NSP1 was higher for lobaric acid (0.720) than for D7, ChemPLP docking yielded a lower score for lobaric acid (77.53) than for D7 (92.31) when the standard OpenBabel 3D structure generation and minimization protocol was used. Notably, docking the Boltz‐2 conformer of lobaric acid directly, without prior OpenBabel minimization, yielded a ChemPLP score of 92.15, closely matching that of D7. This indicates that the initial score discrepancy stemmed from the starting structure used for the docking, probably related to the atypical depsidone scaffold of lobaric acid, a tricyclic system with an ester and an ether linkage forming a central seven‐membered ring. It should also be noted that lobaric acid's activity was demonstrated against CHIKV, not MAYV, NSP1, and that no experimental structure of NSP1 in complex with a small‐molecule ligand is currently available. Both compounds were therefore evaluated under the same protocol. An experimentally determined MAYV NSP1‐D7 binding pose remains the ideal validation for these findings.

Nonetheless, among the evaluated targets, NSP1 emerged as the most promising candidate for D7 binding. The high docking score obtained for the D7–NSP1 complex, relative to the other evaluated targets, is consistent with the antiviral activity observed in biological assays at intracellular stages. To further assess the stability of the predicted binding mode, a 200 ns molecular dynamics simulation of the D7‐NSP1 complex was performed. Throughout the simulation, D7 remained stably bound, with a mean ligand RMSD of 1.78 Å and a mean deviation of 0.39 Å between the ligand and binding‐site centers of mass relative to their initial positions, supporting the persistence of the predicted binding mode over the simulated timescale (Figure S2 – Supporting Information). Considering the role of NSP1 in viral replication [40], this result supports its potential involvement as a molecular target, although the precise mechanism of action remains to be confirmed by target validation studies.

## 4 Conclusion

This study demonstrates that dihydroeugenol‐derived piperazine Mannich bases represent a novel chemical class with selective antiviral activity against MAYV. Compound **D7** emerges as a promising lead molecule with potent inhibition, favorable selectivity, and a post‐entry mechanism of action distinct from existing antiviral agents, with NSP1 identified as a potential molecular target based on the molecular modeling results. These findings contribute to the growing arsenal of candidate molecules targeting neglected tropical arboviruses and provide a foundation for preclinical development of anti‐MAYV therapeutics.

## Author Contributions

Conceptualization, Adriana Cotta Cardoso Reis and Geraldo Célio Brandão; Methodology, Adriana Cotta Cardoso Reis, Diogo Teixeira Carvalho, and Geraldo Célio Brandão; Investigation, Adriana Cotta Cardoso Reis, A.L.S.S, Camila Portruneli, Augusto Vieira Pontes Silva, Saulo Fehelberg Pinto Braga, Igor Rodrigues Lapa, T.B.S., Lucas Lopardi Franco, and Diogo Teixeira Carvalho; Writing Original Draft, Adriana Cotta Cardoso Reis and Diogo Teixeira Carvalho; Writing Review and Editing, Adriana Cotta Cardoso Reis, Saulo Fehelberg Pinto Braga, T.B.S., Diogo Teixeira Carvalho, and Geraldo Célio Brandão; Supervision, Geraldo Célio Brandão; Funding Acquisition, Diogo Teixeira Carvalho, and Geraldo Célio Brandão All authors have read and agreed to the published version of the manuscript.

## Ethics Statement

This article does not contain any studies with human participants or animals performed by any of the authors.

## Conflicts of Interest

The authors declare no conflicts of interest.

## Supporting information


Supporting File 1

Supporting File 2

## Data Availability

The data supporting this study are available from the corresponding author upon reasonable request.
