# Supplementary material for: Eugenol‐Derived Piperazine Mannich Bases as Selective Inhibitors of Mayaro Virus: In Vitro Antiviral Evaluation and Mechanistic Insights
Source: J Med Virol. 2026 Sep 21;98(9):e71158. doi: 10.1002/jmv.71158 (PMC13591657; doi:10.1002/jmv.71158)
Supplement: Supplementary file 1 [file JMV-98-e71158-s001.docx]

**Figure S1 –** Evaluation of the virucidal activity of the piperazine derivative **D7** (23.9 µM) against MAYV [(MOI = 1 (A) and MOI = 10 (B)], by plaque assay in Vero cells. Viral suspensions were pre-incubated with **D7** for 1 h at 37 °C before infection, and viral titers were subsequently determined by plaque assay using 10-fold serial dilutions. Untreated MAYV served as the negative control. Data are expressed as log₁₀ PFU/mL. Experiments were performed in triplicate, and statistical significance was assessed using Student’s *t*-test (95% confidence interval).

MAYV: viral control (MOI = 1 and MOI = 10); **D7** + MAYV: piperazine derivative **D7** (23.9 µM) previously incubated with MAYV suspension (MOI = 1 and MOI = 10) for 1 hour at 37ºC (n = 3), following the titration in Vero cells by the plaque reduction assay.
