# Supplementary material for: Eugenol‐Derived Piperazine Mannich Bases as Selective Inhibitors of Mayaro Virus: In Vitro Antiviral Evaluation and Mechanistic Insights
Source: J Med Virol. 2026 Sep 21;98(9):e71158. doi: 10.1002/jmv.71158 (PMC13591657; doi:10.1002/jmv.71158)
Supplement: Supplementary file 2 [file JMV-98-e71158-s002.docx]

**Figure S2.** Root-mean-square deviation (RMSD) of **D7** relative to its docking-predicted pose and deviation in the center-of-mass (CoM) distance between **D7** and the NSP1 binding-site residues over the 200 ns molecular dynamics simulation. Solid lines represent the 200-frame (4 ns) moving average.
